# Supplementary material for: Vitamin D Supplementation and Allergic Rhinitis: A Systematic Review and Meta-Analysis
Source: Medicina (Kaunas). 2025 Feb 18;61(2):355. doi: 10.3390/medicina61020355 (PMC11857834; doi:10.3390/medicina61020355)
Supplement: Supplementary file 1 [file medicina-61-00355-s001.zip › medicina-3466054-Methods S1 and Figure S1.pdf]

Methods S1. Details of the search strategy

PubMed and Cochrane library

#1
"rhinitis, allergic, seasonal"[MeSH Terms] OR ("rhinitis"[All Fields] AND "allergic"[All Fields] AND "seasonal"[All Fields]) OR "seasonal allergic rhinitis"[All Fields] OR ("hay"[All Fields] AND "fever"[All Fields]) OR "hay fever"[All Fields] OR ("allergie"[All Fields] OR "hypersensitivity"[MeSH Terms] OR "hypersensitivity"[All Fields] OR "allergies"[All Fields] OR "allergy"[All Fields] OR "allergy and immunology"[MeSH Terms] OR ("allergy"[All Fields] AND "immunology"[All Fields]) OR "allergy and immunology"[All Fields]) OR ("allergic"[All Fields] OR "allergical"[All Fields] OR "allergically"[All Fields] OR "allergics"[All Fields] OR "allergization"[All Fields] OR "allergizing"[All Fields]) OR ("rhinitis"[MeSH Terms] OR "rhinitis"[All Fields] OR "rhinitides"[All Fields]) OR "rhinoconjunctivitis"[All Fields] OR ("rhinorrhea"[MeSH Terms] OR "rhinorrhea"[All Fields] OR "rhinorrhoea"[All Fields] OR "rhinorrheas"[All Fields]) OR ("anosmia"[MeSH Terms] OR "anosmia"[All Fields] OR "hyposmia"[All Fields]) OR ("anosmia"[MeSH Terms] OR "anosmia"[All Fields] OR "anosmias"[All Fields]) OR ("nasalance"[All Fields] OR "nasality"[All Fields] OR "nasalization"[All Fields] OR "nasalized"[All Fields] OR "nasally"[All Fields] OR "nose"[MeSH Terms] OR "nose"[All Fields] OR "nasal"[All Fields] OR "nasals"[All Fields])

#2
(((((("vitamin d"[MeSH Terms] OR "vitamin d"[All Fields] OR "ergocalciferols"[MeSH Terms] OR "ergocalciferols"[All Fields] OR ("cholecalciferol"[MeSH Terms] OR "cholecalciferol"[All Fields] OR "cholecalciferols"[All Fields] OR "colecalciferol"[All Fields]) OR ("hydroxycholecalciferols"[MeSH Terms] OR "hydroxycholecalciferols"[All Fields] OR "hydroxycholecalciferol"[All Fields]) OR ("25 hydroxyvitamin d"[Supplementary Concept] OR "25 hydroxyvitamin d"[All Fields] OR "25 hydroxyvitamin d"[All Fields] OR "calcifediol"[MeSH Terms] OR "calcifediol"[All Fields]) OR 25[UID]) AND ("hydroxide ion"[Supplementary Concept] OR "hydroxide ion"[All Fields] OR "oh"[All Fields])) AND ("vitamin d"[MeSH Terms] OR "vitamin d"[All Fields] OR "ergocalciferols"[MeSH Terms] OR "ergocalciferols"[All Fields])) OR 25[UID]) AND ("hydroxide ion"[Supplementary Concept] OR "hydroxide ion"[All Fields] OR "oh"[All Fields])) AND "D"[All Fields]) OR ("calcifediol"[MeSH Terms] OR "calcifediol"[All Fields] OR "calcidiol"[All Fields]) OR ("calcifediol"[MeSH Terms] OR "calcifediol"[All Fields]) OR ("dihydroxycholecalciferols"[MeSH Terms] OR "dihydroxycholecalciferols"[All Fields] OR "dihydroxycholecalciferol"[All Fields]) OR ("1 25 dihydroxyvitamin d"[Supplementary Concept] OR "1 25 dihydroxyvitamin d"[All Fields] OR "1 25 dihydroxyvitamin d"[All Fields]) OR ("calcitriol"[MeSH Terms] OR "calcitriol"[All Fields] OR "calcitriols"[All Fields]) OR ("ergocalciferols"[MeSH Terms] OR "ergocalciferols"[All Fields] OR "ergocalciferol"[All Fields]) OR ("vitamin d deficiency"[MeSH Terms] OR "vitamin d deficiency"[All Fields])

#3
"cohort"[All Fields] OR "cohort s"[All Fields] OR "cohorte"[All Fields] OR "cohorts"[All Fields] OR ("random allocation"[MeSH Terms] OR ("random"[All Fields] AND "allocation"[All Fields]) OR "random allocation"[All Fields] OR "randomization"[All Fields] OR "randomized"[All Fields] OR "random"[All Fields] OR

"randomisation"[All Fields] OR "randomisations"[All Fields] OR "randomise"[All Fields] OR "randomised"[All Fields] OR "randomising"[All Fields] OR "randomizations"[All Fields] OR "randomize"[All Fields] OR "randomizes"[All Fields] OR "randomizing"[All Fields] OR "randomness"[All Fields] OR "randoms"[All Fields])

#1 and #2 and #3

Scopus

#1
(rhinitis and allergic and seasonal) OR (hay and fever) OR (allergie) OR hypersensitivity OR allergies OR allergy OR (allergy and immunology) OR allergic OR allergical OR allergically OR allergics OR allergization OR allergizing OR rhinitis OR rhinitides OR rhinoconjunctivitis OR rhinorrhea OR anosmia OR hyposmia OR anosmia OR anosmias OR nasalance OR nasality OR nasalization OR nasalized OR nasally OR nose OR nasal OR nasals

#2
(vitamin and d) OR ergocalciferols OR cholecalciferol OR cholecalciferols OR hydroxycholecalciferols OR hydroxycholecalciferol OR (25 and hydroxyvitamin and d) OR calcifediol OR calcifediol OR (25 and UID) OR dihydroxycholecalciferols OR (25 and dihydroxyvitamin and d) OR calcitriol OR calcitriols OR ergocalciferols OR ergocalciferol OR (vitamin and d and deficiency)

#3
cohort OR cohorts OR cohorte OR (random AND allocation) OR randomization OR randomized OR random OR randomisation OR randomisations OR randomize OR randomised OR randomising OR randomizations OR randomize OR randomizes OR randomizing OR randomness OR randoms

#1 and #2 and #3

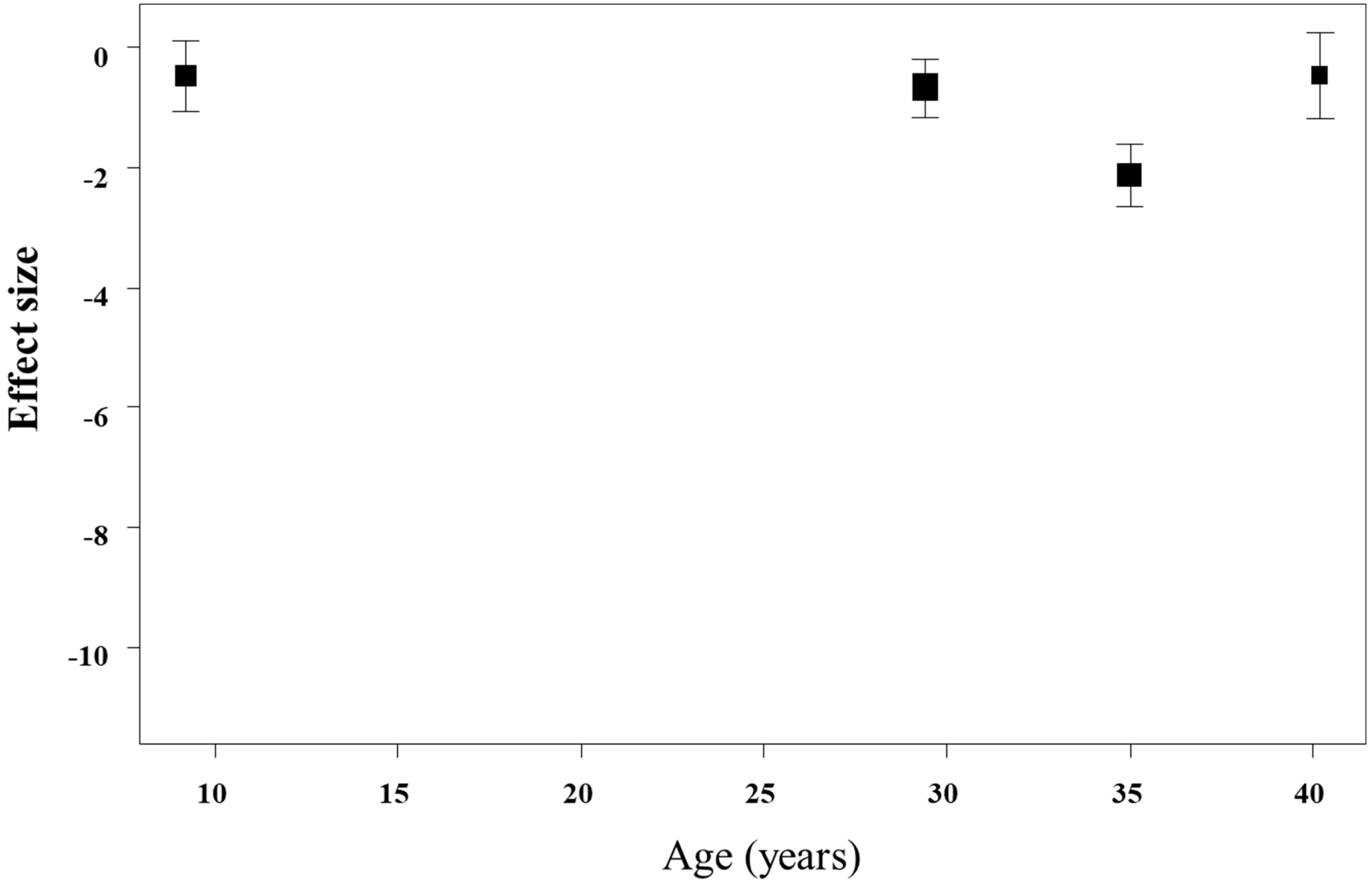

Figure S1-A. Scatter plot showing the association of the average of age in the study vs. SMDs, and the individual studies plotted against a quantitative predictor (X: the average of age in the study, Y: SMDs). The size of the points is drawn proportional to the weight that the studies received in the analysis (with larger points for studies that received more weight).

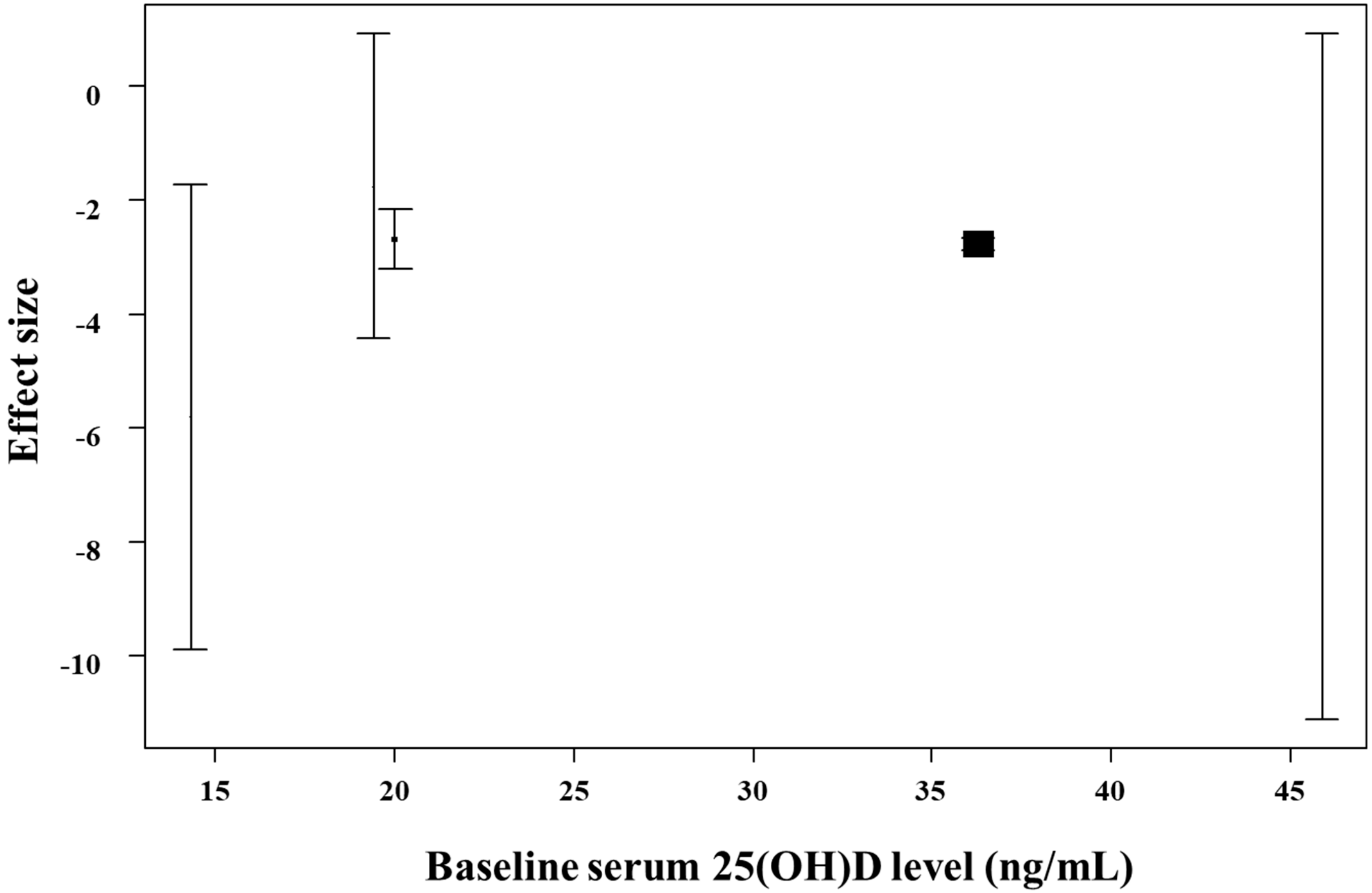

Figure S1-B. Scatter plot showing the association of the baseline serum 25(OH)D levels vs. SMDs, and the individual studies plotted against a quantitative predictor (X: baseline serum 25(OH)D levels, Y: SMDs). The size of the points is drawn proportional to the weight that the studies received in the analysis (with larger points for studies that received more weight).

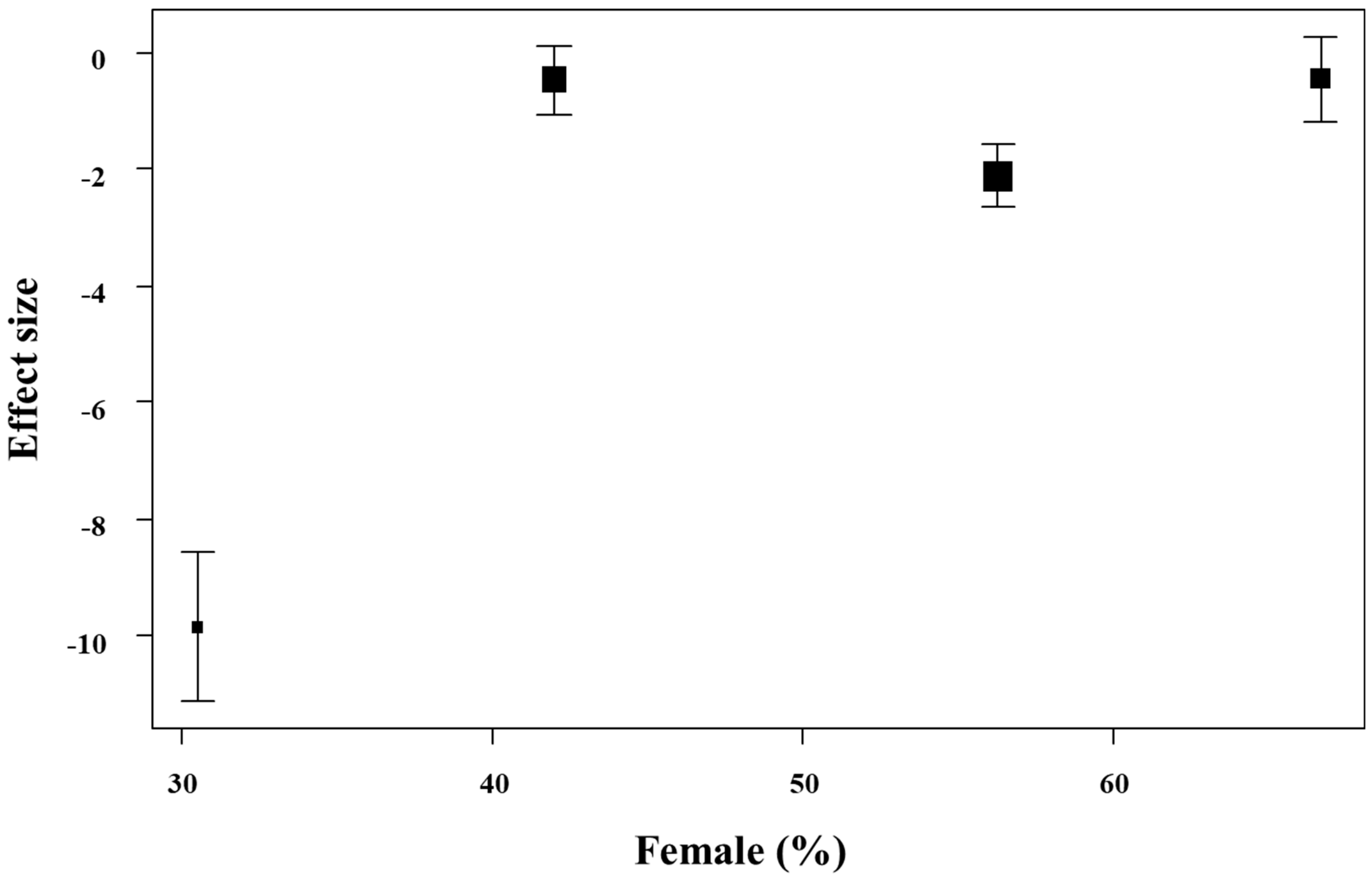

Figure S1-C. Scatter plot showing the association of the proportion of female participants in the study vs. SMDs, and the individual studies plotted against a quantitative predictor (X: the ratio of female in the study, Y: SMDs). The size of the points is drawn proportional to the weight that the studies received in the analysis (with larger points for studies that received more weight).

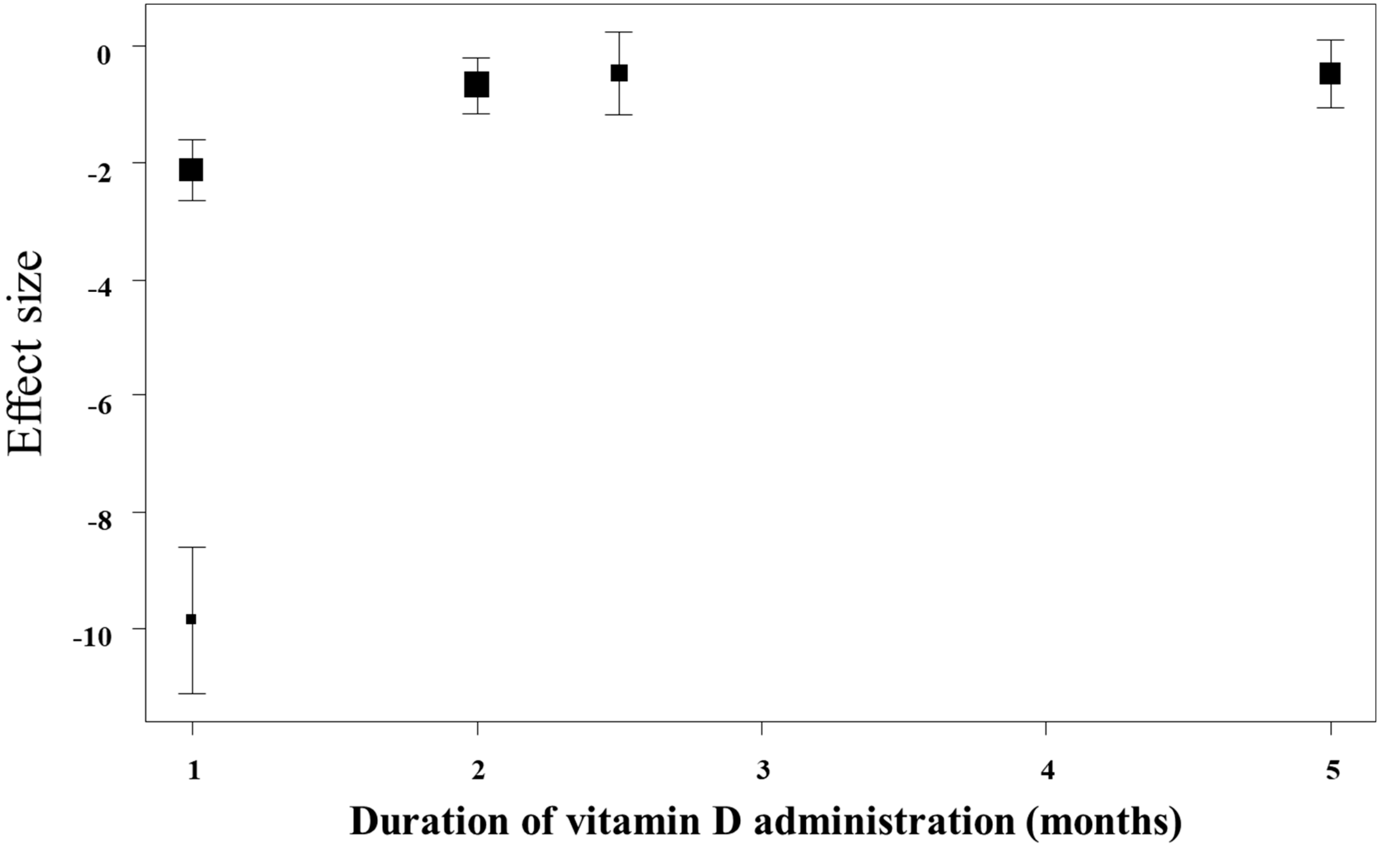

Figure S1-D. Scatter plot showing the association of the duration of vitamin D administration vs. SMDs, and the individual studies plotted against a quantitative predictor (X: the duration of vitamin D administration, Y: SMDs). The size of the points is drawn proportional to the weight that the studies received in the analysis (with larger points for studies that received more weight).
